# Impacts of Industrial Crop Production by Smallholders on Household Food Security in Ghana: Evidence From the Oil Palm Sector

**DOI:** 10.1002/pei3.70172

**Published:** 2026-06-22

**Authors:** Seth Etuah, Robert Aidoo, Kwasi Ohene‐Yankyera, Joyce Haleegoah, Faizal Adams, James Osei Mensah, Awura‐Abena Amoah Etuah, Gifty Boakye Appiah, Steve L. Wiggins

**Affiliations:** ^1^ Department of Agricultural Economics, Agribusiness, and Extension Kwame Nkrumah University of Science and Technology Kumasi Ghana; ^2^ CSIR‐Crop Research Institute Kumasi Ghana; ^3^ Department of Agricultural Economics, and Extension Education Akenten Appiah‐Menka University of Skills Training and Entrepreneurial Development Kumasi Ghana; ^4^ Overseas Development Institute (ODI) London UK

**Keywords:** farm households, food security, industrial crop, oil palm, out grower producers

## Abstract

An increase in industrial crop production has important implications for food security, the environment and rural development in major growing areas of the world. This study assessed the impact of smallholder industrial crop production on the food security of agricultural households. The need for the study arose from the resurgence of debate on the potential negative impacts of industrial crop production on the food security and nutrition of agricultural households in sub‐Saharan Africa. A multi‐stage sampling technique was employed to select 416 farm households, comprising 201 oil palm producers and 215 non‐oil palm producers, from the Ashanti and Central Regions of Ghana. The study adopted a mixed‐method approach. Quantitative data were complemented with key informant interviews and focus group discussions to provide contextual understanding of the findings. The impact evaluation was conducted using both the Endogenous Treatment‐Effect Regression (ETR) and Inverse Probability Weighted Regression Adjustment (IPWRA) techniques. Household food security was measured using the Household Food Insecurity Access Scale and the Household Dietary Diversity Score. In addition, food crop production area was used as an outcome variable to capture household production capacity and potential trade‐offs between oil palm and food crop production. The findings indicate that oil palm production has mixed and context‐specific effects on household food security. It improves dietary diversity in both regions, but its effects on food access differ across locations, improving in the Central Region while worsening in the Ashanti Region. In addition, oil palm production reduces food crop production area due to land reallocation. The results highlight important spatial heterogeneity driven by differences in socioeconomic and production conditions.

## 1 Introduction

Food insecurity remains a pressing challenge in most subregions of Africa, particularly in rural areas where rain‐fed agriculture is the main source of livelihood (FAO et al. 2025). Despite the progress made globally, the number of people unable to afford a healthy diet in Africa increased from 864 million in 2019 to a little above 1 billion in 2024 (i.e., from 64% to 66.6%) (FAO et al. 2025). This increase has been attributed to a growing population, climate change, limited resources, rising food prices, weaker social protection mechanisms, and fewer coping strategies (Delgado et al. 2023; FAO et al. 2025; Mbhenyane et al. 2025). The expansion of industrial crop production has been widely promoted as a pathway for improving rural livelihoods, reducing poverty, and ensuring food and nutrition security in developing countries (Rist et al. 2010; Achterbosch et al. 2014; Kubitza et al. 2018; Edwards 2019; Sibhatu 2019; Balde et al. 2019; Etuah et al. 2020; Khatun et al. 2020; Ayompe et al. 2021; Ruml et al. 2022; Hashmiu et al. 2022).

According to Ayodele and Eshalomi (2010), the cultivation and processing of industrial crops often generate extra cash and jobs along the value chain, thereby boosting the local economy. A study by Hashmiu et al. (2022) confirms that industrial crop cultivation and related activities increase revenue generation and the disposable incomes of farmers, allowing consumption during traditionally lean periods of the year when food insecurity is generally high. In sub‐Saharan Africa, many interventions have been introduced in the sector to enhance productivity and encourage smallholder farmers to transition to the production of industrial crops (e,g., oil palm, cocoa, coffee, cashew) in order to realize their potential benefits. These interventions include rehabilitation of diseased and over‐aged trees, the introduction of hand pollination to boost yield, mass spraying, subsidies on fertilizers and other certified inputs, and training of farmers in the production of other less commercialized tree crops such as cashew, coconut, coffee, and citrus (O'Sullivan et al. 2018; Asante et al. 2023; Amfo et al. 2024).

Despite the reported socioeconomic benefits, there is growing concern that industrial crop production is becoming a leading cause of deforestation, culminating in significant ecological loss (Elmhirst et al. 2015; Vijay et al. 2016; Dislich et al. 2017; Meijaard et al. 2020; Acheampong et al. 2023; Renier et al. 2025). A related study by Sibhatu (2019) in the oil palm sector in Indonesia reports that farm households are increasingly converting rainforests, agroforests, and traditional croplands to oil palm monoculture plantations. According to Vijay et al. (2016), almost all oil palm plantations across the globe are located in areas that were once tropical moist forests. In addition, a recent study by Kalischek et al. (2023) in the cocoa sector reveals that cocoa cultivation is an underlying driver of over 37% of forest loss in protected areas in Côte d'Ivoire and over 13% in Ghana. Other studies on industrial crops have reported a similar trend of land conversion at the expense of environmental sustainability and food crop production (Ruf et al. 2015; Barima et al. 2016; Asubonteng et al. 2018; Carodenuto and Buluran 2021; Renier et al. 2025).

In Ghana, the oil palm sector has emerged as a key industrial crop subsector with increasing participation of smallholder farmers driven by rising global demand, government initiatives and private sector investment (MoFA 2011; Dzanku et al. 2020; Khatun et al. 2020; Ruml et al. 2022). For instance, in 2011, the Ministry of Food and Agriculture (MoFA) commissioned a comprehensive strategic document known as the *Masterplan Study on the Oil Palm Industry* to transform Ghana's palm oil sector into a competitive, commercial and sustainable industry by supporting smallholders through outgrower schemes (MoFA 2011). In the same year, Ghana became the first African country to be approved by the Roundtable on Sustainable Palm Oil (RSPO) for its National Interpretation of the Principles and Criteria for sustainable palm oil production, paving the way for RSPO certification of oil palm growers in Ghana (RSPO 2011, 2022). While oil palm production offers smallholders the opportunity for higher income generation and integration into high‐value markets, its implications for household food security remain poorly understood and empirically contested (Anderman et al. 2014; Sibhatu 2019; Balde et al. 2019; Etuah et al. 2020; Khatun et al. 2020).

Food security, which includes the dimensions of availability, access, utilization and stability, constitutes a central development concern in Ghana, particularly among rural agrarian households. The shift from subsistence‐oriented food crop production to market‐oriented industrial crops such as oil palm may reduce the land, capital and labor allocated to food crops (Anderman et al. 2014; Hansen et al. 2015), thereby potentially undermining food availability at the household level. Moreover, increased reliance on market‐purchased food exposes households to price volatility and market inefficiencies, which may compromise food security stability (Osei et al. 2024). The existing empirical studies on oil palm production have largely focused on productivity (Tan et al. 2012; Rhebergen et al. 2016, 2018; Asravor et al. 2025), adoption of best/sustainable management practices (Anaglo et al. 2014; Atta‐Ankomah and Danso‐Mensah 2022; Cowan and Drewer 2025; Ampofo et al. 2025; Tabe‐Ojong et al. 2023), value chain development and contracts (Ofosu‐Budu and Sarpong 2013; Ruml and Qaim 2020), processing and financial returns (Osei‐Amponsah et al. 2012; Uckert et al. 2015; Sibhatu 2019), policy failures (Asante 2023), and gender roles and women's economic empowerment (Sarku 2016; Etuah et al. 2020).

Studies that explicitly assess the food security impacts of smallholder oil palm production at the household level remain limited. Furthermore, smallholder farmers are heterogenous in terms of resource endowments, farm sizes, market access and institutional support, suggesting that the impacts of oil palm production on food security may not be uniform (Tabe‐Ojong et al. 2023). Against this backdrop, this study aims to provide robust empirical evidence on food security impacts of smallholder oil palm production at the household level, while accounting for potential trade‐offs and heterogeneities. The findings will contribute to ongoing policy debates on the promotion of smallholder industrial crop production and inform strategies aimed at improving livelihoods without compromising food and nutrition security.

## 2 Materials and Methods

### 2.1 Study Area

The study was conducted in the Juaben Municipality in the Ashanti Region and the Upper Denkyira East Municipality in the Central Region of Ghana, as both are recognized as major oil palm producing and processing areas in the country. The selection of these study sites was further informed by the presence of processing companies such as Juaben Oil Mills Limited (JOML) and Twifo Oil Palm Plantations Limited (TOPP), which procure raw materials from smallholder oil palm producers to supplement fruits harvested from their own plantations. The smallholders sampled from the Upper Denkyira East Municipality operate their own lands but have agreements with TOPP to supply portions or all of their harvest to the processing mill in exchange for incentives such as farm inputs, improved seedlings, credit and technical assistance. At the time of the survey, Juaben Oil Mills Limited did not have such organized schemes for its suppliers or smallholder farmers, apart from providing technical assistance. In addition, the oil palm trees in Juaben were relatively older than those in Upper Denkyira East Municipality. This setting therefore presents diverse contexts and two interesting scenarios to investigate the impact of smallholder oil palm production on household food security in Ghana. Besides, the two municipalities are located in different regions with distinct socio‐economic contexts, allowing for data disaggregation.

### 2.2 Sampling and Data Collection

This study employed a multistage sampling technique involving stratified and simple random sampling methods to select communities and smallholder farm households in the study districts. Based on Yamane's (1967) formula, the sample size for the study was computed as 416. Oil palm producers (treatment group) constituted 48.3% of the sample, with 106 respondents from Juaben (Ashanti Region) and 95 from Upper Denkyira East (Central Region), giving a total of 201 respondents. The remaining 51.7% consisted of non‐oil palm producers (control group), comprising 104 respondents from Juaben and 111 from Upper Denkyira East, resulting in a total of 215 respondents. To minimize spillover effects, the non‐oil palm producers were sampled from neighboring communities where oil palm was not cultivated as a major crop. A standardized structured questionnaire was administered in the selected communities by trained enumerators with the use of mobile tablets. This was augmented with key informant interviews and focus group discussions. The mixed‐methods approach allowed for triangulation, stakeholder involvement, and validation of some information provided by respondents.

### 2.3 Method of Data Analysis

#### 2.3.1 Measuring Household Food Security

Household food security was measured using the Household Food Insecurity Access Scale (HFIAS) and the Household Dietary Diversity Score (HDDS). The HFIAS is an analytical tool developed by the Food and Nutrition Technical Assistance (FANTA) III Project/USAID for evaluating the *access* dimension of household food security. It contains nine questions about household members' food availability issues in the month leading up to the survey (Gebreyesus et al. 2015). Each question is ranked on a scale from “zero (No); 1 = Rarely (once or twice in the past four weeks); 2 = Sometimes (three to ten times in the past four weeks); and 3 = Often (more than ten times in the past four weeks)” (Coates et al. 2007). If a household “often” experiences all the stated conditions, then the household's HFIAS score will be 27, which represents the maximum score. The higher the HFIAS score, the greater the extent of food insecurity experienced by the household in terms of food access. The HDDS serves as a proxy for the utilization dimension of food security through dietary quality and diversity. It specifically measures the number of unique food groups consumed by the household over a given period (usually 24 h or 7 days). This study adopted a 7‐day recall period to better capture habitual household food consumption patterns and reduce the influence of daily fluctuations in food intake. The use of food groups instead of the individual foods consumed better reflects the quality of the diet (Cordero‐Ahiman et al. 2021). The food groups used in computing the HDDS are presented below.

A. Cereals; B. Roots and Tubers; C. Vegetables; D. Fruits; E. Meat, poultry; F. Eggs; G. Fish and Seafood; H. Pulses/legumes/nuts; I. Milk and milk products; J. Oils/fats; K. Sugar/honey; L. Miscellaneous.

Based on the food groups, the HDDS is estimated using the mathematical formula:

(1) HDDS=A+B+C+D+E+F+G+H+I+J+K+L

where values from *A* through *L* are either *“0” or “1”*. A higher score suggests a more diverse and higher quality diet. In addition, food crop production area (in acres) was used as an outcome variable to capture household production capacity and potential trade‐offs between oil palm and food crop production.

#### 2.3.2 Measuring the Impact of Oil Palm Production

For the impact assessment, the study adopted the “with and without” approach, whereby comparisons were made between smallholder oil palm‐producing households (treatment group) and non‐oil palm‐producing households (control group). A diagnostic‐driven switching strategy was employed by estimating both the Inverse Probability Weighted Regression Adjustment (IPWRA) model and the Endogenous Treatment‐Effects Regression (ETR) model, after which the most appropriate model for each outcome variable was selected based on the relevant diagnostic tests. This approach was adopted because different outcome variables may respond differently to the underlying assumptions of each estimator, particularly with respect to selection bias, endogeneity, and unobserved heterogeneity. The analysis was initially conducted using the IPWRA model because of its doubly robust property which allows for consistent estimation even when either the treatment‐selection model or the outcome model is misspecified, provided that at least one of the two models is correctly specified (Wooldridge 2002; Ojo et al. 2021). The IPWRA estimator combines the inverse probability weighting approach with the regression adjustment method and is based on the assumption of conditional independence (unconfoundedness), which posits that, conditional on observed covariates, treatment assignment is independent of the potential outcomes (Sseguya et al. 2021). The average treatment effect on the treated (*ATET*) based on the IPWRA estimator can be expressed as:

(2) $$\begin{array}{l} {\text{ATET}}_{\text{IPWRA}}=N^{-1}\sum_{i=1}^{N}\left[\left(\eta_{1}^{*}+\delta_{1}^{*}z_{i}\right)-\left(\eta_{0}^{*}+\delta_{0}^{*}z_{i}\right)\right]\\ \;=\left[\left(\eta_{1}^{*}-\eta_{0}^{*}\right)+\bar{z}\left(\delta_{1}^{*}-\delta_{0}^{*}\right)\right] \end{array}$$

The parameters $\left(\eta_{1}^{*},\delta_{1}^{*}\right)$ are determined through inverse probability‐weighted least squares estimation for the treated group:

(3) $$\min_{\eta_{1\delta 1}}\sum_{i=1}^{N}{\left(Y_{i}-\eta_{1}^{*}-\delta_{1}^{*}z_{1}\right)}^{2}/\hat{p}\left(z,\hat{\lambda }\right)$$

For control group, $\left(\eta_{0}^{*},\delta_{0}^{*}\right)$ is obtained through inverse probability‐weighted least squares estimation of Equation (4).

(4) $$\min_{\eta_{0\delta 0}}\sum_{i=0}^{N}{\left(Y_{i}-\eta_{0}^{*}-\delta_{0}^{*}z_{0}\right)}^{2}/\left(1-\hat{p}\right)\left(z,\hat{\lambda }\right)$$

where $\eta_{t}^{*},\delta_{t}^{*}$ are the estimated inverse probability weighted parameters for control (*t = 0*) and treated (*t = 1*) groups; $\hat{p}=\left(z,\hat{\lambda }\right)$ are the estimated propensity scores; $z_{i}$ denotes observed covariates (explanatory variables); Y refers to the outcome variables (HFIAS, HDDS and food crop production area). The propensity scores are estimated from the selection/adoption equation:

(5) $$T_{i}^{*}=\lambda z_{i}+u_{i}$$

where $T_{i}^{*}$ is the unobserved propensity to adopt or cultivate oil palm and $u_{i}$ is the error term. The observed adoption indicator $\left(T_{i}\right)$ is defined as

(6) $$T_{i}=\left\{\begin{array}{c} 1\\ 0 \end{array}\right.\begin{array}{cc} & \text{if}\;T_{i}^{*}>0\\ & \text{otherwise} \end{array}$$

For outcome variables where diagnostic tests indicated the presence of endogeneity due to unobserved factors, the ETR model was deemed more appropriate, as it explicitly accounts for selection on unobservables through the correlation structure between the treatment and outcome equations. Thus, the ETR framework addresses potential endogeneity arising from self‐selection and unobserved heterogeneity, acknowledging that participation in oil palm cultivation may be non‐random. To specify the ETR model, Equation (5) can be modified to include exclusion restrictions $\left(X_{i}\right)$ as follows:

(7) $$T_{i}^{*}=\gamma X_{i}+\lambda z_{i}+u_{i}$$

where γ is a parameter to be estimated through the probit regession The outcome equation for the ETR is specified as:

(8) $$Y_{i}=\beta z_{i}+\alpha T_{i}+\varepsilon_{i}$$

where $T_{i}$ is the oil palm production indicator; β is a parameter vector; α measures the treatment effect and $\varepsilon_{i}$ is the error term; all other variables are as defined earlier. The ETR model assumes that the error terms from Equations (7) and (8) follow a joint normal distribution:

(9) $$\left(\begin{array}{c} u_{i}\\ \varepsilon_{i} \end{array}\right)∼N\left(\left(\begin{array}{c} 0\\ 0 \end{array}\right),\left(\begin{array}{cc} 1 & \rho \sigma \\ \rho \sigma & \sigma^{2} \end{array}\right)\right)$$

where $u_{i}$ has variance normalized to 1 for identification in Equation (7);$\varepsilon_{i}$ has variance $\sigma^{2}$; and ρ measures the correlation between the two error terms. The parameter ρ captures the degree of endogeneity, if: ρ≠0 then unobserved factors jointly affect both oil palm cultivation and household food security outcomes. Thus, a statistically significant estimate of ρ indicates the presence of correlation between the treatment and outcome equations, suggesting selection on unobservables and a violation of the conditional independence (unconfoundedness) assumption. In such cases, IPWRA estimates may be inefficient or biased if unobserved heterogeneity is not adequately addressed, and a model such as the Endogenous Treatment‐Effects Regression (ETR) becomes more appropriate. The ETR equations are jointly estimated using Full Information Maximum Likelihood (FIML) approach (Maddala 1983; Greene 2000). The log‐likelihood contribution for each observation $\left(L_{j}\right)$ is:

For treated househols $\left(T_{j}=1\right)$

(10) $$\begin{array}{cc} \ln L_{j}\;=\; & \ln\Phi \left(\frac{\gamma X_{i}+\lambda z_{i}+\left(Y_{i}-\beta X_{i}-\alpha \right)\rho /\sigma }{\sqrt{1-\rho^{2}}}\right)\\ & \;-\frac{1}{2}{\left(\frac{Y_{i}-\beta X_{i}-\alpha }{\sigma }\right)}^{2}-\ln\left(\sqrt{2\pi \sigma }\right) \end{array}$$

For treated househols $\left(T_{j}=0\right)$

(11) $$\begin{array}{cc} \ln L_{j}\;=\; & \ln\Phi \left(\frac{-\gamma X_{i}-\lambda z_{i}-\left(Y_{i}-\beta X_{i}\right)\rho /\sigma }{\sqrt{1-\rho^{2}}}\right)\\ & \;-\frac{1}{2}{\left(\frac{Y_{i}-\beta X_{i}}{\sigma }\right)}^{2}-\ln\left(\sqrt{2\pi \sigma }\right) \end{array}$$

where $\Phi \left(\cdot \right)$ is the standard normal cumulative distribution function. The overall log‐likelihood function is

(12) $$\ln L=\sum_{j=1}^{N}\ln L_{j}$$

The parameter estimates are obtained by maximizing the joint log‐likelihood function with respect to: $\left(\gamma ,\lambda ,\beta ,\alpha ,\sigma ,\rho \right)$. The model is estimated using the full information maximum likelihood estimator implemented in the *etregress* command in Stata (StataCorp. 2025). When ρ is statistically insignificant, there is no evidence of selection on unobservables, implying that the conditional independence assumption is more plausible. In this instance, the IPWRA estimator is preferred due to its doubly robust property and efficiency in handling selection on observables.

## 3 Results

### 3.1 Employment Generated by the Industrial Oil Palm Production

**FIGURE 1:**
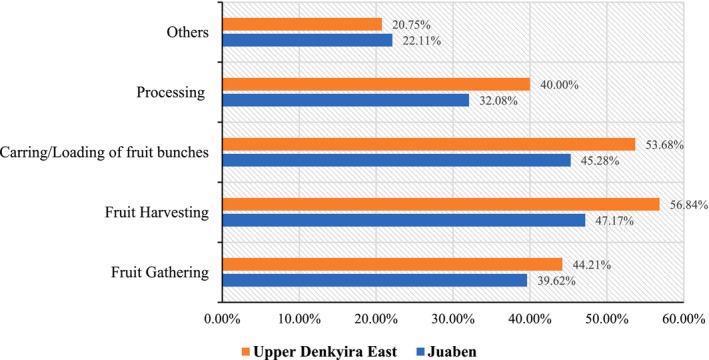
Employment opportunities generated by the industrial oil palm sector.

The employment opportunities generated by industrial oil palm production within farming communities are presented in Figure 1. Harvesting of fresh fruit bunches (FFBs), loose fruit collection, and loading/transportation of palm fruit bunches were identified as the main employment opportunities generated through the promotion of oil palm cultivation in the communities. Nearly half of the smallholder oil palm producers in both Juaben and Upper Denkyira Municipalities confirmed these activities as key livelihood sources for community members, including those not directly engaged in cultivation. The focus group discussions (FGD) and key informant interviews further revealed that loose fruit collectors were mostly older women who gathered fruits that dropped from the bunches and scattered around the palm trees during harvesting. The collected fruits were bagged and subsequently sold to local processors. The loose fruit collectors were able to gather larger quantities during the peak production season (June–September) and relatively smaller quantities during the lean season (November–May), although the price paid per bag remained the same regardless of the season.

In Upper Denkyira East Municipality, most fresh fruit bunch (FFB) harvesters previously worked for the large commercial mill operated by Twifo Oil Palm Plantations Limited (TOPP). However, with the expansion of small‐scale private oil palm plantations within the community, many harvesters found it more profitable to operate independently rather than work for the large oil palm company. In Juaben, most harvesters had been engaged in the activity for many years, as smallholder oil palm cultivation has been practiced in the community for more than a decade. Carriers of fresh fruit bunches (FFBs) were predominantly women, while loaders were mainly young men. Their responsibilities involved gathering harvested bunches from the farms, transporting them to trucks for loading and loading them onto trucks for transportation to factories and individual processing centers. In some cases, carriers also transported the bunches on their heads directly to nearby processing centers. The workers operated with fixed service charges and generally experienced increased workload during the peak production season, as noted by one key informant.

Processing of oil palm was also identified as an important income‐generating activity within the study area. In both municipalities, three main categories of processors were identified: large‐scale processing mills (TOPP and JOM), local mechanized mills operating on a relatively smaller scale compared to the large companies, and individual household traditional processors. The latter group was predominantly dominated by women. The small‐scale mechanized processors mainly relied on custom processing services for individual farmers within their communities for a fee. In contrast, the traditional processors largely depended on manual methods for palm oil extraction. Traditional processing mills were more common in Juaben than in Upper Denkyira East Municipality, largely because oil palm cultivation has a longer history in the former than in the latter. Consequently, these traditional mills emerged primarily to support the small‐scale palm oil processing business within the community. Unlike palm kernel oil processing, which was undertaken exclusively by women, palm oil processing involved a few male processors operating at both commercial and household levels. It further emerged that many individual farmers had begun to realize that owning and managing their own oil palm farms was more economically rewarding than participating in the out‐grower scheme operated by Twifo Oil Palm Plantations Limited (TOPP). In addition, other farm‐based activities such as pruning services, weeding, and fertilizer or herbicide application were also reported by respondents as economic activities emerging from oil palm production.

### 3.2 Crop Area Allocation, Oil Palm Plantation Age and Former Land Use

As shown in Table 1, the average age of the smallholder oil palm plantations was about 15 years in Juaben Municipality and 7 years in Upper Denkyira East Municipality. This indicates that oil palm production has a longer history in the former than in the latter. The sampled smallholders had an average of about 5 acres of land under oil palm cultivation. The average oil palm acreage in Upper Denkyira East Municipality exceeded that of Juaben Municipality by 1.12 acres. Generally, non‐oil palm producers cultivated 0.5 and 0.54 acres more of food crops and other cash crops, respectively, than oil palm producers.

**TABLE 1:** Crop area allocation and oil palm tree age.

Variables	Juabeng		Upper Denkyira East		Pooled Sample		Mean diff. (*t*‐value)
	Oil palm	Non‐oil palm	Oil palm	Non‐oil palm	Oil palm	Non‐oil palm	
Age of oil palm plantations (years)	14.82		7.05	—	11.14	—	—
Oil palm area (acres)	4.23	—	5.41	—	4.79	—	—
Food crop area (acres)	2.15	3.19	2.55	2.49	*2.34*	*2.83*	0.49*** (4.72)
Area for other cash crops (acres)	4.39	5.45	5.84	5.98	*5.19*	*5.73*	0.54 (1.57)

*Note:* Oil palm and non‐oil palm represent oil palm producers and non‐oil palm producers. *** = Not applicable.

**FIGURE 2:**
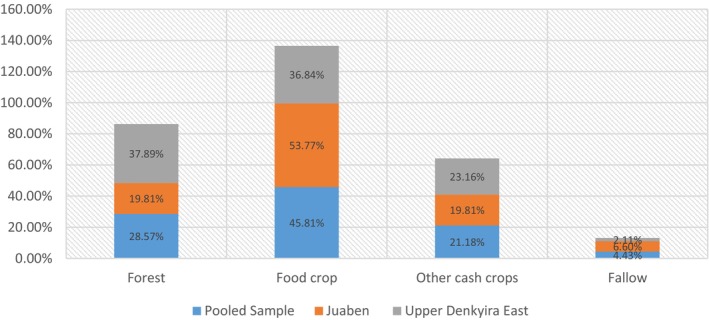
Previous use of oil palm lands.

Figure 2 shows the previous use of lands currently under oil palm cultivation among smallholder farmers across the Municipalities. Most of the lands under oil palm cultivation (45.81%) were formerly used for food crop production. This was followed by forest lands (28.57%) and lands previously used for the cultivation of other cash crops (21.18%). This suggests that the expansion of oil palm production may be associated with the conversion of land previously used for food and other cash crop production with implications for household food availability.

### 3.3 Characteristics of Smallholder Oil Palm Producers and Non‐Oil Palm Producers

The characteristics of smallholder oil palm producers and non‐oil palm producers in the two selected municipalities are presented in Table 2. The summary statistics indicate that smallholder oil palm production is predominantly male‐dominated across both municipalities. However, the proportion of male farmers in the control group was about 9% higher than in the treatment group. The average farmer in the study area was married, with a household size of about five members and a dependency ratio of at least 2. The dependency ratio among oil palm producers was about 0.8 higher than that of their non‐oil palm‐producing counterparts, indicating that treatment households had a relatively higher proportion of dependents (e.g., children and elderly persons), and thus a greater economic burden on working‐age members. The study further found that oil palm producers were more educated than their non‐oil palm‐producing counterparts. Education is expected to enhance farmers' ability to understand and adopt improved technologies, thereby improving productivity and welfare. The farmers were generally in the middle‐age category (40–51 years), with those in the treatment group being about 2 years older on average than their counterparts in the control group. Generally, less than 35% of the sampled smallholder farmers had access to credit, which could constrain their ability to use improved inputs and enhance productivity.

**TABLE 2:** Characteristics of smallholder oil palm producers and non‐oil palm producers.

Variable	Juaben municipality			Upper Denkyira East Municipality			Pooled sample		
	Non‐oil palm	Oil palm		Non‐oil palm	Oil palm		Non‐oil palm	Oil palm	
	Mean	Mean	Diff.	Mean	Mean	Diff.	Mean	Mean	Diff.
Gender (male = 1)	0.73	0.69	0.04	0.80	0.67	0.13**	0.77	0.68	0.09**
Marital status (married = 1)	0.76	0.68	0.08	0.78	0.68	0.10	0.78	0.68	0.10**
Age (years)	49.06	51.39	2.32*	40.83	42.58	1.75	41.88	44.29	2.41**
Education (formal education = 1)	0.79	0.86	0.07	0.70	0.80	0.10	0.74	0.83	0.09**
Household size (number of people in the household)	5.46	6.45	0.99**	5.44	5.14	0.30	5.45	5.84	0.39
Dependency ratio	1.70	2.46	0.76	2.15	2.97	0.82	1.93	2.70	0.77*
Adult labor (people of working age in the household)	2.71	3.02	0.31	2.71	2.80	0.09	2.71	2.92	0.21
Formal training in farming (training = 1)	0.44	0.46	0.02	0.47	0.44	0.03	0.46	0.45	0.01
Oil palm promotion exposure (yes = 1)	0.44	0.58	0.14*	0.48	0.57	0.09	0.46	0.57	0.11**
Distance to an oil palm milling company (km)	37.53	14.81	22.72***	30.77	26.87	3.90***	34.04	20.51	13.53***
FBO membership (member = 1)	0.18	0.33	0.15***	0.15	0.26	0.11***	0.16	0.30	0.14***
Land tenure system (own/family land = 1)	0.75	0.77	0.02	0.68	0.88	0.20***	0.69	0.78	0.09**
Native (native = 1)	0.63	0.60	0.03	0.55	0.67	0.12*	0.59	0.64	0.05
Off‐farm income (yes = 1)	0.29	0.27	0.20	0.28	0.36	0.08	0.29	0.31	0.02
Cultivation of other cash crops (yes = 1)	0.84	0.64	0.20***	0.87	0.79	0.08	0.85	0.71	0.14***
Credit access (yes = 1)	0.37	0.35	0.02	0.29	0.32	0.03	0.33	0.34	0.01
Remittance (receive remittances = 1)	0.38	0.42	0.04	0.38	0.37	0.01	0.37	0.39	0.02
Cultivation of food crops (yes = 1)	0.84	0.64	0.28***	0.67	0.69	0.02	0.74	0.61	0.13***
Livestock (rearing of livestock = 1)	0.38	0.27	0.11	0.35	0.39	0.04	0.36	0.33	0.03
Distance to nearest market (km)	1.79	1.68	0.11	1.69	2.12	0.43	1.74	1.89	0.15
Total farm size (acres)	7.91	9.12	1.21*	8.09	12.38	4.29***	8.01	10.66	2.66***
District (Juaben = 1)	—	—	—	—	—	—	0.48	0.53	0.05

*Note:* Standard errors in parentheses.

* *p* < 0.

** *p* < 0.05.

*** *p* < 0.01.

A typical household in the study areas had about three (3) members in the working‐age group (18–60 years), irrespective of the treatment and the municipality. At least 44% of the respondents had received formal training in farming and had been exposed to oil palm promotion campaigns by non‐governmental organizations (NGOs) and other government agencies. As expected, the treatment group had benefited from more of such campaigns than their control counterparts. The majority of the respondents (at least 68%) farmed on their own/family lands. However, the proportion of farmers using own/family land was slightly higher among those in the treatment group across the two municipalities. On average, the smallholder farmers' residencies were 2 km from the nearest agricultural markets, and this could have transportation cost implications.

An average oil palm farmer lived about 15 and 27 km from the nearest oil palm milling company in Juaben and Upper Denkyira East municipalities respectively. Nonetheless, non‐oil palm producers were located more than 30 km away from such companies. This suggests that proximity to oil palm processing company with guaranteed market could be an incentive for farmers to engage in oil palm production. Overall, about 30% of the oil palm producers were members of farmer‐based associations compared to only 16% of the non‐oil palm producers. The proportion of non‐oil palm producers engaged in food crop production exceeded that of oil palm producers by about 13%, suggesting that non‐oil palm producers are relatively more involved in food crop cultivation. In Juaben, about 63% of non‐oil palm producers were natives compared to 60% of oil palm producers. In Upper Denkyira East Municipality, the proportion of natives was 55% among non‐oil palm producers and 67% among oil palm producers. Overall, the results indicate modest differences in nativity status between oil palm and non‐oil palm producers, with a statistically significant higher proportion of natives observed among oil palm producers in Upper Denkyira East Municipality.

The proportion of respondents receiving remittances was about 2% higher in the treatment group than in the control group. In upper Denkyira East Municipality, about 36% of oil palm producers were engaged in off‐farm income‐generating activities such as petty trading and other local businesses, compared to 28% of non‐oil palm producers. However, in Juaben Municipality, non‐oil palm producers were more engaged in off‐farm income‐generating activities.

This could increase their household income and purchasing power, resulting in improved food/nutrition security. Moreover, about 39% of oil palm producers in Upper Denkyira East Municipality were engaged in livestock rearing, compared to 35% of non‐oil palm producers. Nonetheless, the situation in Juaben Municipality was the opposite, where non‐oil palm producers were more engaged in livestock rearing than oil palm producers. Livestock is perceived as an indicator of wealth and serves as a form of security against periods of food insecurity. The proportion of non‐oil palm producers engaged in the production of other cash crops such as cocoa, citrus, cashew, and rubber was 84% and 87% in Juaben and Upper Denkyira East Municipalities, respectively. The corresponding figures for oil palm producers were 64% and 79% in the two municipalities, respectively. This suggests that non‐oil palm producers are more engaged in the production of other cash crops. The total farm size, measured as the total acreage of land owned or cultivated by the household for agricultural activities, ranged from a minimum of about 8 acres among non‐oil palm producers in Juaben to a maximum of about 12 acres among oil palm producers in Upper Denkyira East. Overall, oil palm producers had about 3 acres more land on average than non‐oil palm producers.

### 3.4 Food Security Indicators

#### 3.4.1 Household Food Insecurity Access Scale (HFIAS)

The household food insecurity Access Scale (HFIAS) scores for oil palm producers and non‐oil palm producers are presented in Table 3. As emphasized earlier, the HFIAS score measures the degree of household food insecurity in terms of access (Coates et al. 2007). The mean HFIAS score among oil palm producers (4.74) was higher than that of non‐oil palm producers (2.20) in the Juaben Municipality, suggesting comparatively higher levels of food insecurity among oil palm‐producing households. In contrast, the situation in the Upper Denkyira East Municipality differed, with oil palm producers recording a mean HFIAS score that was 1.81 points lower than that of non‐oil palm producers, implying relatively lower levels of food insecurity among oil palm‐producing households in the municipality.

**TABLE 3:** Household food insecurity access scale (HFIAS).

	Juaben			Upper Denkyira East			Pooled sample		
	Oil palm	Non‐oil palm	Diff.	Oil palm	Non‐oil palm	Diff.	Oil palm	Non‐oil palm	Diff.
Mean HFIAS	4.74	2.20	2.54***	1.86	3.67	1.81***	3.38	2.96	0.42
Std. dev.	4.56	2.98	—	2.52	3.86	—	3.53	4.00	—
COV	0.96	1.35	—	1.35	1.06	—	1.18	1.19	—

*Note:* *** denotes statistical significance at 1%; COV represents coefficient of variation; Std. dev. is the standard deviation. A higher HFIAS score indicates a higher level of household food insecurity.

**FIGURE 3:**
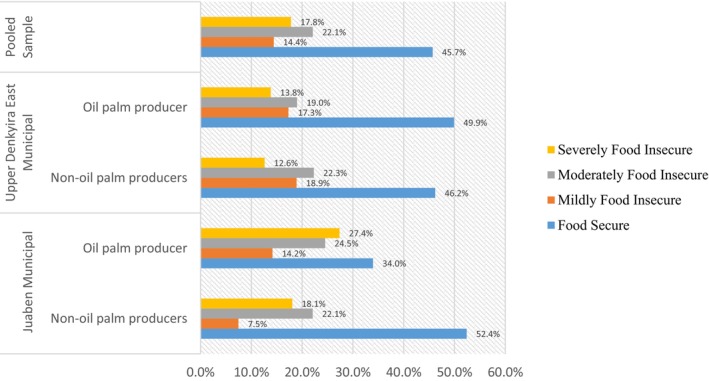
Proportion of households under various food insecurity (access) categories.

Generally, the large variations around the mean scores suggest substantial disparities in food security status among households, indicating that while some households were relatively food secure, others experienced severe food insecurity in both groups (Cov≈1.2). In addition, the proportions of households in the various food insecurity (access) categories among the control and treatment groups are presented in Figure 3. Using the Household Food Insecurity Access Prevalence (HFIAP) indicator, households were categorized as *food secure, mildly food insecure*, *moderately food insecure or severely food insecure*. Any household that responded affirmatively to more severe conditions and/or experienced those conditions more frequently was classified as increasingly food insecure (Coates et al. 2007).

Overall, about 46% of the sampled households were classified as food secure, while about 18% fell within the severely food insecure category. About 50% of the smallholder oil palm‐producing households in Upper Denkyira East Municipality fell within the food secure category, compared to 34% in Juaben Municipality. Respondents in the severely food insecure category accounted for about 27% of the treatment group and 18% of the control group in Juaben Municipality. However, the proportions of respondents in the severely food insecure category were almost the same for both groups in Upper Denkyira East Municipality.

#### 3.4.2 Household Dietary Diversity Score (HDDS)

Table 4 presents the HDDS for the sampled smallholder households in both groups across the municipalities. The HDDS helps assess the economic ability of a household to consume a variety of food groups (Swindale and Bilinsky 2006). Overall, oil palm‐producing households recorded an HDDS of 8.86, compared to 7.85 among non‐oil palm‐producing households. This suggests that oil palm producers generally consumed more diverse diets than their counterparts who did not cultivate oil palm. A similar pattern was observed across the various municipalities, although oil palm producers in Upper Denkyira East Municipality exhibited greater dietary diversity than those in Juaben Municipality. However, there were no households with extremely high dietary diversity or extreme dependence on a limited range of food groups.

**TABLE 4:** Average household dietary diversity score (HDDS).

	Juaben			Upper Denkyira East			Pooled sample		
	Oil palm	Non‐oil palm	Diff.	Oil palm	Non‐oil palm	Diff.	Oil palm	Non‐oil palm	Diff.
Mean HDDS	8.46	7.36	1.1***	8.95	8.31	0.64**	8.69	7.85	0.84***
Std. dev.	1.59	1.90	—	1.57	1.59	—	1.59	1.82	—
COV	0.19	0.26	—	0.17	0.19	—	0.18	0.23	—

*Note:* ** and *** denote statistical significance at 5% and 1% respectively.

### 3.5 Determinants of Food Security and Food Crop Production Area

As indicated earlier, this study estimated both the Endogenous Treatment‐Effect Regression (ETR) and Inverse Probability Weighted Regression Adjustment (IPWRA) models to evaluate the impact of smallholder oil palm production on food security and food crop production area. The appropriate model, based on the diagnostic tests, was subsequently discussed. Table 5 presents the results of the Sargan (1958) and Basmann (1960) overidentification tests used to assess the validity of distance to the oil palm milling company and oil palm promotion exposure as instruments (exclusion restrictions) in the ETR model. The test results were statistically insignificant (*p* > 0.05), indicating that the null hypothesis of valid instruments could not be rejected. This suggests that the instruments satisfy the exclusion restriction and are appropriate for identifying the treatment effect.

**TABLE 5:** Validity of the instruments.

Outcome variable	Sargan test		Basmann test		Interpretation
	Statistic	*p*	Statistic	*p*	
HFIAS	0.00902	0.9243	0.00865	0.9259	Instruments valid
HDDS	0.16686	0.6829	0.16011	0.6891	Instruments valid
Food crop acreage	0.62092	0.4307	0.59644	0.4399	Instruments valid

None of the Wald chi‐square tests of independence in the estimated ETR models were statistically significant in Juaben Municipality, indicating that the null hypothesis of no correlation between the treatment and outcome equations $\left(\rho =0\right)$ could not be rejected (see Table A3 in the Appendix). This further suggests that there was no strong evidence of endogeneity arising from self‐selection and unobserved heterogeneity, and hence the IPWRA results were prioritized due to their double robustness and higher efficiency. On the other hand, the test was statistically significant $\left(\rho \neq 0\right)$ at the 5% level for the ETR model estimating the impact of oil palm production on food crop acreage, but not for the other outcome variables in Upper Denkyira East Municipality. As such, the ETR results were prioritized for that analysis.

As shown in Table A4 (in the Appendix), the estimated correlation $\left(\rho \right)$ was negative (−0.940), indicating that unobservables that increased food crop production acreage were associated with unobservables that reduced participation in oil palm production. The ETR estimates indicate that smallholder farmers engaged in off‐farm income‐generating activities had about 0.43 acres lower food crop acreage. In addition, members of farmer‐based organizations recorded about 0.64 acres lower food crop acreage. An increase in total land size resulted in a 0.06‐acre increase in food crop production acreage. Moreover, an increase in the distance from farming communities to agricultural markets reduced food crop cultivation by 0.093 acres, reflecting transaction cost effects on food crop production and sales. Farmers who owned or cultivated family land had 1.03 acres lower food crop acreage, likely due to greater concentration on cash crops.

**FIGURE A1:**
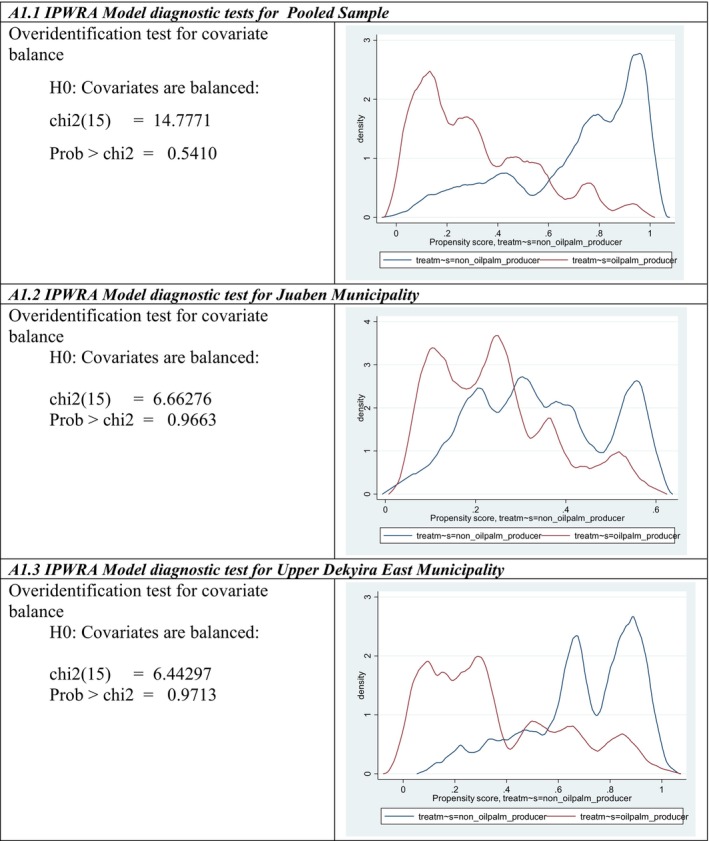
Covariate balance and overlap assessment for the IPWRA model.

Tables 6 and 7 present the maximum likelihood estimates of the IPWRA model, indicating the determinants of participation in oil palm production, food security and food crop production acreage. The covariate balance tests produced *p*‐values ranging from 0.54 to 0.97, indicating that the null hypothesis that the covariates were balanced could not be rejected. The overlap graphs showed substantial common support between the treatment and control groups (see Figure A1 in the Appendix). In addition, there was no evidence of multicollinearity among the covariates used in the IPWRA and ETR estimations (see Tables A1 and A2 in the Appendix). The results indicate that male farmers were more likely to cultivate oil palm than their female counterparts, particularly in Juaben Municipality. However, married smallholder farmers had a lower propensity to cultivate oil palm. An increase in household size increased the likelihood of cultivating oil palm in Juaben Municipality but reduced it in Upper Denkyira East Municipality. Membership in farmer‐based organizations promoted the cultivation of oil palm across the municipalities. Natives in Juaben municipality were more likely to produce oil palm compared to the settlers. An increase in the distance from the farming communities to the oil palm milling companies reduced the likelihood of smallholder farmers engaging in oil palm production regardless of the municipality. In line with a priori expectations, an increase in total household land size improved the likelihood of cultivating oil palm (Crop cultivation dummies and crop‐specific land areas were excluded from the regression models to avoid post‐treatment bias, as they are likely to be endogenous outcomes of oil palm participation and reflect land reallocation decisions. Including them would condition on post‐treatment variables and potentially bias the estimated treatment effects by blocking part of the causal pathway. Total farm size, measured as household land endowment, is retained to capture differences in land availability across households).

**TABLE 6:** Estimates of inverse‐probability‐weighted regression adjustment (IPWRA)—Juaben.

Variables	Household Food Insecurity Access Scale (HFIAS)			Household dietary score (HDDS)			Food crop acreage (FCA)		
	Treatment status	Non‐oil palm producer	Oil palm producer	Treatment status	Non‐oil palm producer	Oil palm producer	Treatment Status	Non‐oil palm producer	Oil palm Producer
Gender	0.831*	1.615*	−0.931	0.831*	1.913***	1.118***	0.831*	−1.460***	0.247
	(0.491)	(0.971)	(1.187)	(0.449)	(0.569)	(0.417)	(0.470)	(0.488)	(0.287)
Married	−1.232*	−1.052*	1.784	−1.232*	−0.287	−1.016**	−1.232*	−0.795	0.288
	(0.677)	(0.630)	(1.391)	(0.677)	(0.549)	(0.441)	(0.677)	(0.769)	(0.325)
Age	0.030	−0.001	−0.040	0.030	−0.032*	−0.037***	0.030	0.053**	0.0233*
	(0.031)	(0.040)	(0.056)	(0.031)	(0.017)	(0.014)	(0.031)	(0.023)	(0.014)
Education	0.094	−1.948*	−2.550*	0.094	−2.144***	0.155	0.094	1.806**	0.027
	(0.699)	(1.115)	(1.418)	(0.699)	(0.661)	(0.435)	(0.699)	(0.853)	(0.395)
Household size	0.256**	0.178*	0.080	0.256**	−0.064	0.084*	0.256**	0.185**	−0.049
	(0.121)	(0.104)	(0.169)	(0.121)	(0.075)	(0.049)	(0.121)	(0.074)	(0.042)
Credit	−0.228	−1.236	−1.034	−0.228	−0.269	0.764***	−0.228	0.463	0.986***
	(0.540)	(0.951)	(0.879)	(0.540)	(0.612)	(0.294)	(0.540)	(0.424)	(0.319)
Off‐farm	−0.569	−0.651	−1.500*	−0.569	−0.305	0.765**	−0.569	−1.468***	0.537*
	(0.531)	(0.599)	(0.877)	(0.531)	(0.624)	(0.342)	(0.531)	(0.445)	(0.276)
FBO	0.832*	−2.637**	−2.082**	0.832*	−0.875*	−0.040	0.832*	−1.301**	0.0114
	(0.500)	(1.176)	(0.888)	(0.422)	(0.499)	(0.394)	(0.491)	(0.588)	(0.307)
Livestock rearing	—	−4.325***	−4.804***	—	−1.806***	0.460*	—	0.326	0.313
		(0.689)	(0.779)		(0.340)	(0.238)		(0.466)	(0.285)
Remittance	—	−0.0384	0.351	—	0.353	−0.132	—	1.600**	0.433*
		(0.918)	(0.907)		(0.638)	(0.326)		(0.684)	(0.219)
Native	0.732*	−0.848	0.816	0.732*	0.906*	0.208	0.732*	−0.827*	0.133
	(0.418)	(0.844)	(0.832)	(0.417)	(0.535)	(0.311)	(0.414)	(0.425)	(0.266)
Total land size	−0.044	0.0433	−0.140*	−0.044	0.166*	0.0232	−0.042	0.380***	0.074***
	(0.048)	(0.099)	(0.083)	(0.048)	(0.098)	(0.0377)	(0.048)	(0.0488)	(0.026)
Distance to market	0.101	−0.332	0.381	0.101	−1.544***	0.0903	0.101	−0.556*	0.074
	(0.171)	(0.572)	(0.328)	(0.171)	(0.218)	(0.0923)	(0.171)	(0.317)	(0.103)
Land tenure system	0.004	−0.945	−0.507	0.004	−1.137*	0.856**	0.004	−1.165*	0.170
	(0.645)	(1.620)	(1.138)	(0.645)	(0.644)	(0.361)	(0.645)	(0.754)	(0.290)
Distance to oil palm mill	−0.100**	—	—	−0.100***	—	—	−0.100***	—	—
	(0.034)			(0.033)			(0.033)		
Oil palm promotion exposure	0.538	—	—	0.538	—	—	0.538	—	—
	(0.482)			(0.482)			(0.482)		
Constant	−0.316	7.566**	11.46***	−0.316	12.64***	7.823***	−0.316	−1.462	−0.894
	(1.832)	(3.837)	(4.006)	(1.832)	(1.279)	(0.918)	(1.832)	(1.545)	(1.148)
Observations	210			210			210		

*Note:* Robust standard errors in parentheses.

* *p* < 0.1.

** *p* < 0.05.

*** *p* < 0.01.

**TABLE 7:** Estimates of inverse‐probability‐weighted regression adjustment (IPWRA)‐Upper Denkyera East.

Variables	Household Food Insecurity Access Scale (HFIAS)			Household Dietary Score (HDDS)			Food Crop Acreage (FCA)		
	Treatmen statust	Non‐oil palm producer	Oil palm producer	Treatment status	Non‐oil palm producer	Oil palm producer	Treatment status	Non‐oil palm	Oil palm producer
Gender	−0.006	0.180	−1.280*	−0.006	−1.110**	−0.478	−0.006	1.572***	−0.536*
	(0.603)	(1.172)	(0.795)	(0.603)	(0.519)	(0.517)	(0.603)	(0.530)	(0.302)
Married	−0.206	−0.858	−1.44**	−0.206	1.182**	0.004	−0.206	−1.656***	0.480
	(0.569)	(1.208)	(0.718)	(0.569)	(0.522)	(0.541)	(0.569)	(0.459)	(0.420)
Age	−0.025	−0.0901**	0.002	−0.025	−0.026*	−0.015	−0.025	−0.026*	−0.015
	(0.019)	(0.0431)	(0.031)	(0.019)	(0.016)	(0.016)	(0.019)	(0.016)	(0.018)
Education	0.373	−3.236***	−0.976*	0.373	0.767**	0.112	0.373	−0.403	−0.099
	(0.459)	(0.892)	(0.600)	(0.459)	(0.385)	(0.305)	(0.459)	(0.387)	(0.220)
Household size	−0.138*	−0.306**	0.020	−0.138*	0.177**	0.322***	−0.138*	0.041	−0.021
	(0.071)	(0.152)	(0.091)	(0.0718)	(0.078)	(0.065)	(0.0718)	(0.081)	(0.062)
Credit	0.241	0.485	−0.820*	0.241	−0.615*	−0.472*	0.241	0.431	−0.310
	(0.394)	(0.799)	(0.503)	(0.394)	(0.372)	(0.257)	(0.394)	(0.420)	(0.279)
Off‐farm	0.261	−0.425	−0.358	0.261	0.212	0.276	0.261	−1.612***	0.009
	(0.451)	(0.949)	(0.518)	(0.451)	(0.445)	(0.294)	(0.451)	(0.406)	(0.324)
FBO	0.950**	2.127*	−0.225	0.950**	0.797*	−0.507*	0.950**	2.432***	−0.213*
	(0.461)	(1.196)	(0.669)	(0.461)	(0.570)	(0.316)	(0.461)	(0.799)	(0.142)
Livestock rearing	—	1.019	0.005	—	−0.023	−0.691**	—	−0.252	0.124
		(0.901)	(0.445)		(0.423)	(0.306)		(0.397)	(0.287)
Remittance	—	0.235	−0.909*	—	0.362	0.646**	—	−0.179	0.089
		(1.007)	(0.554)		(0.425)	(0.324)		(0.561)	(0.244)
Native	0.123	−1.116	0.478	0.123	0.190	0.075	0.123	0.214	−0.292
	(0.405)	(0.906)	(0.503)	(0.405)	(0.474)	(0.305)	(0.405)	(0.365)	(0.333)
Total land size	0.241***	0.0596	−0.070	0.241***	0.0179	0.045**	0.241***	0.432***	0.123***
	(0.044)	(0.105)	(0.062)	(0.0449)	(0.042)	(0.020)	(0.044)	(0.048)	(0.029)
Distance to market	0.120**	0.118	−0.054	0.120**	−0.323*	0.016	0.092***	−0.188	−0.025
	(0.054)	(0.442)	(0.054)	(0.0545)	(0.194)	(0.042)	(0.536)	(0.156)	(0.024)
Land tenure system	0.891*	0.685	−1.325*	0.891*	−0.530	0.286	0.891*	−0.467	−1.032**
	(0.53)	(0.934)	(0.709)	(0.536)	(0.447)	(0.354)	(0.536)	(0.412)	(0.440)
Distance to oil palm	−0.092***	—	—	−0.0917***			−0.092***	—	—
mill	(0.033)			(0.0333)			(0.033)		
Oil palm promotion	0.295	—	—	0.295			0.295	—	—
exposure	(0.359)			(0.359)			(0.359)		
Constant	0.438	10.93***	5.047***	0.438	8.431***	7.625***	0.438	0.953	3.104***
	(1.550)	(2.359)	(1.579)	(1.550)	(1.357)	(0.949)	(1.550)	(1.025)	(0.946)
Observations	206			206			206		

*Note:* Robust standard errors in parentheses.

* *p* < 0.1.

** *p* < 0.05.

*** *p* < 0.01.

Interestingly, an increase in distance from the farming communities to agricultural markets positively influenced the probability of cultivating oil palm in Juaben Municipality. Besides, smallholder farmers who worked on their own or family land with secure tenure were more likely to cultivate oil palm as a long‐term investment (see Table 7).

On the determinants of food security, male‐headed oil palm‐producing households recorded lower HFIAS scores than their female counterparts across the municipalities, suggesting better access to food. However, among non‐oil palm‐producing households in Juaben Municipality, male‐headed households experienced higher food insecurity, possibly due to greater dependence on market‐based food access and lower engagement in subsistence food production. Married smallholder farmers experienced lower food insecurity in terms of access, regardless of municipality or treatment status. Furthermore, a year increase in the age of the farmer reduced the HFIAS score by 0.009 points among non‐oil palm producers in Upper Denkyira East Municipality. Besides, farmers with formal education experienced lower food insecurity across the municipalities and treatment groups, emphasizing the crucial role of education in household livelihood decision‐making in the sampled communities.

For non‐oil palm producers, an increase in household size reduced HFIAS scores by 0.31 points in Upper Denkira East Municipality, while it increased the score by 0.178 points in Juaben Municipality. Engagement in off‐farm income‐generating activities and access to credit significantly reduced HFIAS scores among oil palm producers in Juaben and Upper Denkyira East Municipalities, respectively. Similarly, membership in farmer‐based organizations and livestock rearing generally lowered food insecurity in Juaben Municipality. Oil palm producers who owned/used family land or received remittances recorded lower food insecurity in terms of access, particularly in Upper Denkyira East Municipality (Tables 6 and 7).

As shown in Table 6, an increase in household size, engagement in off‐farm income‐generating activities, access to credit, male household headship, livestock rearing, and secure land tenure (own/family land) were associated with higher dietary diversity scores (HDDS) among oil palm producers in Juaben Municipality. On the other hand, membership in farmer‐based organizations, remittances, an increase in total land size, and larger household size significantly improved HDDS among oil palm producers in Upper Denkyira East Municipality, suggesting regional heterogeneity in livelihood structures and household resource allocation patterns (Table 7). For non‐oil palm producers, formal education, age of the farmer, membership in farmer‐based organizations, livestock rearing, land tenure system, and distance to the nearest agricultural market negatively influenced household dietary diversity scores in Juaben Municipality. Nonetheless, total land size, being a native, and male household headship increased HDDS among these households. A similar pattern was observed among their counterparts in Upper Denkyira East Municipality, with the exception that education, membership in farmer‐based organizations, and marital status had positive effects on HDDS.

For farmers in Juaben Municipality, the IPWRA estimates revealed that total land size, remittances, engagement in off‐farm income‐generating activities, access to credit and age of the farmers positively influenced food crop production acreage among oil palm producers (Table 6). For non‐oil palm producers, male household headship, membership in farmer‐based organizations, being a native, and land tenure system (own/family land) had negative effects on food crop production acreage. However, household size, land size, and education positively influenced food crop production acreage among these households. The pooled sample analyses revealed a similar pattern of influence for all the covariates (see Tables A5 and A6 in the Appendix).

### 3.6 Implications of Oil Palm Production for Food Security and Food Crop Area

The impacts of smallholder oil palm production on food security indicators such as the Household Food Insecurity Access Scale (HFIAS) and Household Dietary Diversity Score (HDDS), as well as food crop production area, are presented in Table 8. In impact assessment, it is the average treatment effect on the treated (ATET) that is of substantive interest (Wang et al. 2017). The ATET measures the effect of an intervention on the outcomes of the participants. In a policy context, the interest is on whether the intervention has been beneficial for the participants rather than all individuals or non‐participants (Heckman and Vytlacil 2001). Since only the ETR estimates for food crop production area in Upper Denkyira East Municipality exhibited significant endogeneity, the ATET estimates from the IPWRA model were prioritized for the remaining outcome variables and for food crop production area in Juaben Municipality. For Upper Denyira East Municipality, participation in oil palm production reduced HFIAS scores by 1.903 points and increased HDDS by 0.754 points, indicating improved food security in terms of access and greater dietary diversification. In addition, oil palm producers cultivated 1.327 more acres of food crops than non‐oil palm producers after adjusting for endogeneity. For Juaben Municipality, oil palm production increased HDDS by 0.762. However, oil palm producers cultivated about 1.5 acres less food crop area and experienced higher food insecurity (2.282) in terms of access. The pooled sample estimates exhibits as similar pattern of influence as Juaben Muncipality.

**TABLE 8:** Average treatment effects on the treated (ATET) based on ETR & IPWRA.

Outcome variables	Juaben		Upper Denkyera East		Pooled sample	
	ETR	IPWRA	ETR	IPWRA	ETR	IPWRA
HFIAS	2.442*** (0.677)	2.282*** (0.574)	−2.808 (3.447)	−1.903*** (0.583)	1.794*** (0.606)	0.414 (0.426)
HDDS	0.901** (0.414)	0.762** (0.334)	0.408 (1.753)	0.754** (0.295)	0.863** (0.350)	0.719*** (0.228)
Food crop acreage	−1.341*** (0.303)	−1.484*** (0.363)	1.327*** (0.554)	1.804*** (0.401)	−1.027*** (0.278)	−1.431*** (0.220)

*Note:* Robust standard errors in parentheses.

** *p* < 0.05.

*** *p* < 0.01.

## 4 Discussion

The promotion of smallholder oil palm production in rural communities has resulted in the emergence of various stakeholders beyond direct cultivators and large commercial oil mills. These include small‐scale processors, estate workers, commercial mill workers, harvesters, carriers of harvested bunches, loose fruit gatherers, loaders, and transporters (Figure 1). Thus, smallholder oil palm cultivation has a profound multiplier effect in terms of employment generation and value addition activities in the two municipalities studied. Oil palm cultivation has a longer history in Juaben Municipality than in Upper Denkyira East Municipality, with plantations in the former being about twice as old as those in the latter (Table 1). However, the average smallholder oil palm plantation in Upper Denkyira East was about 1.17 acres larger than that in Juaben. In Upper Denkyira East, oil palm producers also cultivated larger areas of food crops, whereas the reverse was observed in Juaben, suggesting differences in land use patterns and production priorities across the two municipalities. Generally, non‐oil palm producers allocated more land to food crop cultivation, suggesting that participation in oil palm production may compete with food crop production, particularly in areas with limited land availability (Table 1). The findings further indicate that lands currently under oil palm cultivation were previously used for food crops, other cash crops, forest, or fallow land, in order of importance, suggesting that oil palm expansion largely occurred through the conversion of existing agricultural lands rather than forested areas (Figure 2). Based on the Household Food Insecurity Access Prevalence (HFIAP) indicator (Coates et al. 2007), 27.4% of the oil palm producers in Juaben Municipality could be classified as severely food insecure, compared to 13.8% in Upper Denkyira East Municipality, indicating that food insecurity was more severe in the former than in the latter. Interestingly, more than half of the non‐oil palm producers (52.4%) in Juaben Municipality were food secure compared to only 34% among their oil palm‐producing counterparts (Figure 3). The key informant interviews confirmed that most oil palm producers, particularly in Juaben Municipality, did not produce sufficient food crops to sustain their households throughout the year and therefore relied on the market during certain periods of the year, thereby affecting household food access. This finding is consistent with Osei et al. (2024), who reported that most smallholder farmers in Ghana are net food consumers and depend on the market for food, especially during the lean season, thereby exposing them to food price volatility and food insecurity.

Moreover, the results generally indicate that male farmers were more likely to cultivate oil palm compared to their female counterparts. This is not unexpected because, culturally, cash crop production in Ghana and Sub‐Saharan Africa at large is dominated by men (Mensah and Fosu‐Mensah 2020). Besides, women tend to have limited access to productive resources such as land, labor, credit, and extension services, which constrain their ability to engage in cash or industrial crop production (Etuah et al. 2020). An increase in household size and, for that matter, labor availability raised the likelihood of producing oil palm. Since oil palm cultivation is labor‐intensive, all other things being equal, an increase in household labor availability could serve as an incentive to participate in oil palm cultivation (Tables 6 and 7). As expected, an increase in the distance from farming communities to oil palm milling companies discouraged farmers from engaging in oil palm production due to the anticipated difficulty in accessing ready markets and technical assistance provided by such companies. Natives were more likely to cultivate oil palm, as oil palm plantations are often considered long‐term family assets and a form of inheritance for younger generations. Married farmers were less likely to engage in oil palm production, possibly due to competing household financial responsibilities and risk considerations associated with long‐term investment crops (Etuah et al. 2020). Membership in farmer‐based organizations (FBOs) promoted smallholder oil palm cultivation in the study area. Such organizations often provide capacity‐enhancing workshops on the production of different crops, opportunities for sharing experiences among farmers from diverse backgrounds, and access to production‐related information and networks. These opportunities could stimulate farmers' interest in cultivating cash crops such as oil palm.

Smallholder farmers engaged in livestock rearing were more diversified in food consumption and had improved access to food (Tables 6 and 7). This is expected because income generated from livestock production enhances household purchasing power and, consequently, reduces food insecurity. In addition, some livestock can be slaughtered for household consumption during periods of food shortages or sold to purchase other food items. Livestock is therefore perceived as a form of wealth and serves as a buffer against periods of food insecurity. This finding is consistent with those of previous studies (Sindhu et al. 2008; Abdulla 2015). Farmers who were formally educated, older, married, and male experienced improved access to food but did not necessarily diversify their food consumption. The positive effects of these characteristics on food access could be attributed to their influence on income generation, access to productive resources, farming experience, labor availability and household decision‐making. These factors may enhance households' capacity to secure sufficient food throughout the year and reduce vulnerability to food insecurity. However, a household may have adequate access to food while still consuming monotonous diets. This suggests that although these characteristics may improve households' ability to secure food, they do not automatically translate into more diverse dietary patterns (Tables 6, 7, and A6). On the other hand, engagement in off‐farm income‐generating activities, access to credit, and secure land tenure (own/family land) improved dietary diversity. This is because off‐farm income sources increase household purchasing power, enabling households to access a wider variety of foods from the market. Similarly, access to credit relaxes liquidity constraints, allowing households to smooth consumption and purchase diverse and nutrient‐rich foods, especially during lean seasons. Secure land tenure enhances farmers' confidence in long‐term investment and income stability, which improves overall livelihood security and facilitates better dietary choices (Tables 6 and 7). In line with expectations, farmers with larger total land size, those who receive remittances, have access to credit, and engage in off‐farm income‐generating activities tend to cultivate more acres of food crops. This is because larger landholdings provide greater production capacity and allow for expansion of cultivated area. Remittances increase available household capital for investment in agriculture, while access to credit relaxes liquidity constraints and enables the purchase of inputs such as seeds, fertilizers, and hired labor. Similarly, off‐farm income‐generating activities diversify household income sources, improving financial capacity to expand farm operations. Collectively, these factors enhance farmers' ability to increase food crop acreage and improve household food production.

The impact assessment revealed that oil palm production improved household dietary diversity scores and reduced food crop production area in the two municipalities. This is because participation in oil palm production increases household income and market dependence, enabling households to purchase a wider variety of foods, while simultaneously encouraging land reallocation from food crops to oil palm cultivation. However, oil palm production improved food access in Upper Denkyira Municipality while it reduced it in Juaben Municipality (Table 8). Focus group discussions and key informant interviews revealed that oil palm producers in Upper Denkyira Municipality had relatively younger plantations that allowed intercropping with food crops, which was not commonly practiced in Juaben Municipality. Moreover, the average oil palm plantation size in Upper Denkyira East was about 1.2 acres larger than in Juaben, which could translate into higher output and income. It was also revealed that smallholder oil palm producers in Upper Denkyira East received input support (fertilizer, herbicides, etc.) from oil palm milling companies in exchange for selling their produce to them. These benefits likely reduced production costs, increased yields, improved income, and ultimately enhanced food access. This evidence shows that the impact of oil palm on food security is context‐specific and cannot be generalized (Tabe‐Ojong et al. 2023).

## 5 Conclusions

This study assessed the impact of smallholder oil palm production on household food security and food crop production using data obtained from a survey of 416 farm households, comprising 201 oil palm producers and 215 non‐oil palm producers, from the Ashanti Region (Juaben Muncipality) and Central Region (Upper Denkyira East Municipality) of Ghana. The impact evaluation was conducted using both the Endogenous Treatment‐Effect Regression (ETR) and Inverse Probability Weighted Regression Adjustment (IPWRA) techniques. The findings suggest that oil palm production has mixed and context‐specific effects on food security outcomes. In general, oil palm production improved household dietary diversity in both municipalities, indicating enhanced food consumption variety. However, its effects on food access (HFIAS) differed across locations, with improvements observed in Upper Denkyira East Municipality, while food access deteriorated in Juaben Municipality. In addition, oil palm production was associated with a reduction in food crop production area, suggesting land reallocation from food crops to oil palm cultivation. The study further revealed important heterogeneity in the determinants of food security and production decisions across municipalities. Factors such as education, household size, access to credit, off‐farm income, land tenure security, and membership in farmer‐based organizations played significant but varying roles in shaping food security outcomes. The evidence from qualitative interviews also confirmed that differences in plantation age, intercropping practices, input support systems, and plantation size contributed to the observed spatial variation in outcomes. Overall, the results demonstrate that the welfare effects of oil palm production are not uniform but depend on local conditions, institutional support, and production systems. Future studies could use longitudinal data to better capture the long‐term and dynamic impacts of oil palm production on household food security and welfare outcomes.

## Funding

The field data collection was funded by the Overseas Development Institute (ODI), United Kingdom (Ref: F01529).

## Conflicts of Interest

The authors declare no conflicts of interest.

## Data Availability

The data that support the findings of this study are available from Overseas Development Institute (ODI), United Kingdom (UK). Restrictions apply to the availability of these data, which were used under license for this study. Data are available from the author(s) with the permission of Overseas Development Institute (ODI), United Kingdom (UK).

## Appendix A

**TABLE A1:** Variance inflation factor (VIF) for variables in outcome equation.

Variable	VIF	1/VIF
Gender	2.01	0.498
Marital status	2.03	0.494
Age	1.15	0.868
Education	1.11	0.905
Household size	1.19	0.838
Credit	1.03	0.971
Off‐farm	1.06	0.944
FBO	1.14	0.874
Livestock rearing	1.09	0.915
Remittance	1.16	0.865
Native	1.16	0.865
Total land size	1.14	0.878
Distance to nearest market	1.02	0.978
Land tenure	1.20	0.836
Municipality	1.11	0.900
Mean VIF	1.24	

**TABLE A2:** Variance inflation factor (VIF) for variables in treatment equation.

Variable	VIF	1/VIF
Gender	1.99	0.503
Marital status	2.02	0.495
Age	1.13	0.884
Education	1.14	0.877
Household size	1.19	0.840
Credit	1.03	0.971
Off‐farm	1.06	0.943
FBO	1.13	0.886
Distance to oil palm milling comp.	1.09	0.917
Native	1.17	0.855
Total land size	1.12	0.893
Distance to nearest market	1.02	0.980
Land tenure	1.18	0.847
Municipality	1.12	0.893
Mean VIF	1.23	

**TABLE A3:** Estimates of the endogenous treatment regression (ETR) models for Juaben.

Variable	Household Food Insecurity Access Scale (HFIAS)		Household dietary score (HDDS)		Food crop acreage (FCA)	
	Oil palm production	HFIAS	Oil palm production	HDDS	Oil palm production	FCA
Gender	0.525 (0.351)	0.245 (0.794)	0.502 (0.358)	0.630* (0.339)	0.529 (0.353)	0.086 (0.278)
Married	−1.003*** (0.367)	0.494 (0.822)	−0.994*** (0.373)	−0.551* (0.342)	−0.994*** (0.369)	0.259 (0.279)
Age	0.0267* (0.015)	−0.047* (0.027)	0.0283* (0.015)	−0.005 (0.014)	0.027* (0.015)	0.012* (0.007)
Education	0.187 (0.383)	−1.278* (0.719)	0.206 (0.371)	0.274 (0.327)	0.179 (0.371)	0.037 (0.269)
Household size	0.158*** (0.045)	0.093 (0.083)	0.159*** (0.046)	0.061* (0.038)	0.155*** (0.045)	0.021 (0.032)
Credit	−0.296 (0.275)	−0.407 (0.469)	−0.306 (0.275)	0.169 (0.236)	−0.306 (0.275)	0.476** (0.202)
Off‐farm	−0.598** (0.292)	−0.700 (0.466)	−0.624** (0.298)	0.657** (0.257)	−0.615** (0.291)	−0.002 (0.203)
FBO	0.695** (0.299)	−1.580*** (0.548)	0.682** (0.294)	0.109 (0.290)	0.698** (0.298)	0.234 (0.257)
Livestock rearing	—	−3.021*** (0.441)	—	−0.179 (0.272)	—	0.193 (0.228)
Remittance	—	−0.375 (0.577)	—	0.266 (0.265)	—	0.160 (0.211)
Native	0.412* (0.224)	−0.104 (0.516)	0.429* (0.267)	0.436* (0.249)	0.444* (0.275)	0.044 (0.210)
Total land size	−0.017 (0.027)	−0.027 (0.058)	−0.017 (0.027)	0.016 (0.030)	−0.017 (0.027)	0.175*** (0.027)
Distance to nearest market	0.091 (0.102)	0.087 (0.257)	0.099 (0.101)	−0.052 (0.098)	0.098 (0.102)	0.008 (0.093)
Land tenure system	0.069 (0.335)	−0.382 (0.636)	0.089 (0.337)	0.713** (0.278)	0.072 (0.333)	0.195 (0.213)
Distance to oil palm mill	−0.102*** (0.009)	—	−0.103*** (0.009)	—	−0.103*** (0.009)	—
Oil palm promotion exposure	0.315 (0.261)	—	0.305 (0.261)	—	0.315 (0.264)	—
Treatment (ATET)	—	2.442*** (0.677)	—	0.901** (0.414)	—	−1.341*** (0.303)
Constant	0.023 (1.001)	6.881*** (1.799)	−0.066 (0.998)	5.788*** (0.825)	0.002 (0.991)	0.307 (0.635)
/athrho	0.096 (0.155)		0.071 (0.243)		0.048 (0.186)	
/lnsigma	1.225** (0.057)		0.496*** (0.043)		0.308*** (0.057)	
Rho	0.096 (0.154)		0.071 (0.242)		0.048 (0.185)	
Sigma	3.404*** (0.196)		1.643*** (0.070)		1.360*** (0.077)	
Lambda	0.328 (0.526)		0.328 (0.526)		0.065 (0.253)	
Wald test of indep. eqns. (rho = 0): chi^2^(1) *(p‐value)*	0.39 (0.534)		0.08 (0.771)		0.07 (0.797)	

*Note:* Robust standard errors in parentheses.

* *p* < 0.1.

** *p* < 0.05.

*** *p* < 0.01.

**TABLE A4:** Estimates of endogenous treatment regression (ETR)—Upper Denkyera East.

Variable	Household Food Insecurity Access Scale (HFIAS)		Household dietary score (HDDS)		Food crop acreage (FCA)	
	Oil palm production	HFIAS	Oil palm production	HDDS	Oil palm production	FCA
Gender	0.162 (0.398)	0.006 (0.939)	0.089 (0.446)	0.041 (0.402)	−0.127 (0.278)	0.192 (0.411)
Married	−0.355 (0.390)	0.694 (0.864)	−0.167 (0.326)	0.129 (0.415)	−0.212 (0.289)	−0.063 (0.392)
Age	−0.009 (0.012)	−0.016 (0.029)	−0.011 (0.013)	−0.004 (0.012)	−0.011 (0.009)	−0.006 (0.013)
Education	0.255 (0.254)	−2.013*** (0.705)	0.288 (0.257)	0.454* (0.271)	0.303 (0.210)	−0.332 (0.298)
Household size	−0.050 (0.054)	0.0195 (0.109)	−0.085* (0.046)	0.132** (0.055)	−0.044 (0.043)	0.071 (0.066)
Credit	0.043 (0.275)	0.121 (0.556)	0.143 (0.231)	−0.351* (0.219)	0.128 (0.199)	−0.361 (0.304)
Off‐farm	0.181 (0.231)	−0.832* (0.516)	0.220 (0.249)	0.312 (0.252)	0.342* (0.202)	−0.428* (0.267)
FBO	0.719** (0.290)	−0.574 (1.017)	0.545** (0.258)	0.366 (0.470)	0.604** (0.280)	−0.644* (0.389)
Livestock rearing	—	−0.398 (0.467)	—	−0.196 (0.215)	—	0.166 (0.195)
Remittance	—	−0.594 (0.530)	—	0.508** (0.236)	—	−0.003 (0.207)
Native	0.124 (0.240)	−0.545 (0.597)	0.075 (0.235)	0.278 (0.264)	0.052 (0.194)	0.0348 (0.309)
Total land size	0.131*** (0.028)	−0.234** (0.116)	0.139*** (0.023)	0.075 (0.079)	0.138*** (0.0228)	0.057* (0.034)
Distance to nearest market	0.069** (0.027)	−0.083 (0.093)	0.071** (0.029)	0.016 (0.051)	0.042** (0.018)	−0.093*** (0.030)
Land tenure system	0.442* (0.265)	−0.685 (0.839)	0.539* (0.308)	0.234 (0.357)	0.297 (0.232)	−1.026*** (0.369)
Distance to oil palm mill	−0.036* (0.022)	—	−0.052** (0.021)	—	−0.026*** (0.009)	—
Oil palm promotion exposure	0.092 (0.234)	—	0.103 (0.239)	—	0.027 (0.112)	—
Treatment (ATET)	—	2.808 (3.448)	—	−0.408 (1.754)	—	1.327*** (0.555)
Constant	−0.478 (1.253)	7.174*** (1.725)	0.009 (1.130)	6.531*** (0.645)	−0.460 (0.606)	2.074*** (0.703)
/athrho	−0.884 (0.807)		0.384 (0.767)		−1.740*** (0.431)	
/lnsigma	1.285*** (0.219)		0.405*** (0.125)		0.618*** (0.109)	
Rho	−0.708 (0.422)		0.366 (0.664)		−0.940*** (0.050)	
Sigma	3.614*** (0.793)		1.499*** (0.188)		1.856*** (0.203)	
Lambda	−2.561 (2.002)		0.548 (1.059)		−1.745*** (0.274)	
Wald test of indep. eqns. (rho = 0): chi^2^(1) *(p‐value)*	*1.20 (0.273)*		*0.25 (0.616)*		16.26*** (0.000)	

*Note:* Robust standard errors in parentheses. The italics are *p*‐values associated with the Wild test of independence of equations.

* *p* < 0.1.

** *p* < 0.05.

*** *p* < 0.01.

**TABLE A5:** Estimates of endogenous treatment regression (ETR) model—Pooled sample.

Variable	Household Food Insecurity Access Scale (HFIAS)		Household dietary score (HDDS)		Food crop acreage (FCA)	
	Oil palm production	HFIAS	Oil palm production	HDDS	Oil palm production	FCA
Gender	0.169 (0.215)	0.107 (0.582)	0.152 (0.216)	0.459* (0.254)	0.151 (0.214)	0.116 (0.211)
Married	−0.547** (0.216)	0.543 (0.574)	−0.526** (0.219)	−0.163 (0.284)	−0.520** (0.217)	0.101 (0.206)
Age	0.003 (0.008)	−0.030* (0.018)	0.002 (0.008)	−0.007 (0.008)	0.002 (0.008)	−0.007 (0.007)
Education	0.301* (0.187)	−1.582*** (0.494)	0.314* (0.196)	0.280 (0.192)	0.313* (0.195)	−0.101 (0.167)
Household size	0.031 (0.028)	0.063 (0.062)	0.031 (0.028)	0.096*** (0.028)	0.031 (0.029)	0.003 (0.028)
Credit	−0.068 (0.162)	−0.183 (0.357)	−0.067 (0.161)	−0.108 (0.160)	−0.064 (0.162)	0.105 (0.152)
Off‐farm	−0.071 (0.181)	−0.815** (0.349)	−0.0753 (0.181)	0.459*** (0.174)	−0.072 (0.182)	−0.126 (0.152)
FBO	0.500*** (0.191)	−0.876** (0.422)	0.486** (0.192)	0.071 (0.205)	0.486** (0.191)	0.110 (0.200)
Livestock rearing	—	−1.751*** (0.347)	—	0.007 (0.171)	—	0.247* (0.163)
Remittance	—	−0.364 (0.411)	—	0.396** (0.177)	—	0.150 (0.148)
Native	0.183 (0.162)	−0.402 (0.378)	0.171 (0.162)	0.388** (0.179)	0.169 (0.162)	0.077 (0.159)
Total land size	0.070*** (0.016)	−0.119*** (0.042)	0.071*** (0.016)	0.023 (0.021)	0.071*** (0.016)	0.193*** (0.020)
Municipality (Juaben)	−0.192 (0.151)	0.598* (0.371)	−0.222 (0.147)	−0.773*** (0.165)	−0.223 (0.148)	0.525*** (0.153)
Distance to nearest market	0.045* (0.026)	−0.033 (0.064)	0.042* (0.026)	−0.016 (0.026)	0.043* (0.026)	−0.013 (0.023)
Land tenure system	0.083 (0.188)	−0.303 (0.438)	0.059 (0.188)	0.371* (0.193)	0.077 (0.190)	−0.207 (0.172)
Distance to oil palm mill	−0.074*** (0.007)	—	−0.074*** (0.006)	—	−0.074*** (0.007)	—
Oil palm promotion exposure	0.074 (0.148)	—	0.073 (0.150)	—	0.080 (0.152)	—
Treatment (ATET)	—	1.794*** (0.606)	—	0.863** (0.350)	—	−1.027*** (0.278)
Constant	0.723 (0.524)	6.760*** (1.198)	0.811 (0.526)	6.615*** (0.482)	0.787 (0.528)	1.172*** (0.421)
/athrho	−0.193* (0.109)		−0.085 (0.157)		0.029 (0.138)	
/lnsigma	1.259*** (0.105)		0.462*** (0.033)		0.343*** (0.039)	
Rho	−0.190* (0.105)		−0.085 (0.155)		0.029 (0.131)	
Sigma	3.524*** (0.158)		1.586*** (0.053)		1.409*** (0.056)	
Lambda	−0.670* (0.380)		−0.135 (0.248)		0.042 (0.195)	
Wald test of indep. eqns. (rho = 0): chi^2^(1) *(p‐value)*	3.08* (0.079)		0.30 (0.585)		0.05 (0.830)	

*Note:* Robust standard errors in parentheses.

* *p* < 0.1.

** *p* < 0.05.

*** *p* < 0.01.

**TABLE A6:** Estimates of inverse‐probability‐weighted regression adjustment (IPWRA)‐pooled sample.

Variables	Household Food Insecurity Access Scale (HFIAS)			Household dietary score (HDDS)			Food crop acreage (FCA)		
	Treatment	Non‐oil palm	Oil palm	Treat‐ment	Non‐oil palm	Oil palm	Treat‐ment	Non‐oil palm	Oil palm
Gender	0.254	1.029	0.038	0.254	0.421	0.504	0.254	0.633	−0.089
	(0.371)	(0.752)	(0.846)	(0.371)	(0.477)	(0.332)	(0.371)	(0.619)	(0.253)
Married	−0.926**	−0.420	0.115	−0.926**	−0.280	−0.213	−0.926**	−0.866*	0.591**
	(0.380)	(0.851)	(0.886)	(0.380)	(0.499)	(0.373)	(0.380)	(0.458)	(0.261)
Age	0.004	0.025	−0.031	0.004	0.001	−0.027**	0.004	−0.005	−0.002
	(0.015)	(0.022)	(0.032)	(0.015)	(0.015)	(0.011)	(0.015)	(0.015)	(0.012)
Education	0.584*	−2.943***	−1.488**	0.584*	0.766**	0.049	0.584*	0.260	−0.084
	(0.353)	(0.882)	(0.696)	(0.353)	(0.380)	(0.261)	(0.353)	(0.373)	(0.190)
Household size	0.055	−0.170**	0.059	0.055	0.144***	0.131***	0.055	0.075	−0.027
	(0.052)	(0.075)	(0.098)	(0.052)	(0.051)	(0.036)	(0.052)	(0.058)	(0.033)
Credit	−0.117	−0.539	−0.458	−0.117	−0.953**	0.062	−0.117	0.206	0.158
	(0.282)	(0.568)	(0.517)	(0.282)	(0.464)	(0.203)	(0.282)	(0.347)	(0.214)
Off‐farm	−0.134	−1.799***	−0.511	−0.134	0.193	0.476**	−0.134	−0.955***	0.026
	(0.326)	(0.528)	(0.491)	(0.326)	(0.424)	(0.226)	(0.326)	(0.297)	(0.202)
FBO	0.863***	0.949*	−1.345**	0.863***	0.833***	−0.154	0.863***	0.339	−0.039
	(0.327)	(0.565)	(0.570)	(0.327)	(0.288)	(0.257)	(0.327)	(0.434)	(0.241)
Livestock rearing	—	−0.948*	−2.196***	—	−0.105	−0.157	—	0.585*	0.060
		(0.542)	(0.469)		(0.387)	(0.238)		(0.347)	(0.221)
Remittance	—	0.145	−0.211	—	0.274	0.390	—	0.458	0.141
		(0.701)	(0.601)		(0.372)	(0.239)		(0.451)	(0.190)
Native	0.290	−0.452	−0.031	0.290	−0.014	0.267	0.290	−0.107	−0.114
	(0.283)	(0.565)	(0.520)	(0.283)	(0.460)	(0.236)	(0.283)	(0.341)	(0.221)
Total land size	0.127***	0.007	−0.058	0.127***	0.048	0.0374	0.066	−0.280*	−0.006
	(0.028)	(0.080)	(0.057)	(0.028)	(0.046)	(0.025)	(0.048)	(0.158)	(0.024)
Distance to market	0.066	0.197	0.036	0.066	−0.436**	0.035	0.072	0.061	−0.346
	(0.048)	(0.378)	(0.061)	(0.048)	(0.177)	(0.030)	(0.330)	(0.410)	(0.270)
Land tenure system	0.072	0.266	−0.079	0.072	−0.295	0.515**	0.127***	0.372***	0.118***
	(0.330)	(0.859)	(0.646)	(0.330)	(0.349)	(0.258)	(0.028)	(0.033)	(0.022)
Juaben	−0.322	−1.045*	2.670***	−0.322	−0.827**	−0.451**	−0.322	0.120	0.018
	(0.260)	(0.581)	(0.572)	(0.260)	(0.352)	(0.219)	(0.260)	(0.379)	(0.206)
Distance to oil	−0.127***	—	—	−0.127***	—	—	−0.127***	—	—
Palm mill	(0.013)			(0.012)			(0.012)		
Oil palm promotion	0.148	—	—	0.148	—	—	0.148	—	—
Exposure	(0.260)			(0.260)			(0.260)		
Constant	1.286	5.587***	6.486***	1.286	7.301***	8.004***	1.286	−0.215	1.305*
	(0.942)	(1.768)	(1.923)	(0.942)	(0.970)	(0.711)	(0.942)	(1.045)	(0.746)
Observations	416			416			416		

*Note:* Robust standard errors in parentheses.

* *p* < 0.1.

** *p* < 0.05.

*** *p* < 0.01.
